# Perioperative comparison between robotic-assisted and freehand total knee arthroplasty: A quasi-randomized controlled trial

**DOI:** 10.1007/s00132-025-04709-5

**Published:** 2025-09-01

**Authors:** Filippo Migliorini, Luise Schäfer, Jens Schneider, Andrea Maria Nobili, Daniel Kämmer, Nicola Maffulli, Andreas Bell

**Affiliations:** 1 Department of Trauma and Reconstructive Surgery, University Hospital of Halle, Martin-Luther University Halle-Wittenberg, Ernst-Grube-Street 40, 06097 Halle (Saale), Germany; 2 Department of Orthopaedic and Trauma Surgery, Eifelklinik St.Brigida, Kammerbruschstr. 8, 52152 Simmerath, Germany; 3 Department of Orthopaedic and Trauma Surgery, Academic Hospital of Bolzano (SABES-ASDAA), via Lorenz Böhler 5, 39100 Bolzano, Italy; 4 Department of Life Sciences, Health, and Health Professions, Link Campus University, Via del Casale di San Pio V, 00165 Rome, Italy; 5 Department of Trauma and Orthopaedic Surgery, Faculty of Medicine and Psychology, University La Sapienza, 00185 Rome, Italy; 6 Faculty of Medicine, School of Pharmacy and Bioengineering, Keele University, ST4 7QB Stoke on Trent, UK; 7 Centre for Sports and Exercise Medicine, Barts and the London School of Medicine and Dentistry, Mile End Hospital, Queen Mary University of London, E1 4DG London, UK

**Keywords:** Arthroplasty outcomes, Surgical accuracy, Implant alignment, Soft tissue balance, Functional recovery, Ergebnisse der Endoprothetik, Chirurgische Genauigkeit, Implantationsgenauigkeit, Weichteilspannung, Funktionelle Erholung

## Abstract

**Background:**

The advent of navigation, followed by robotics in knee prosthetic surgery aims, among other things, to enhance the alignment of components and to improve the control of stress forces (i.e., weight, gravity, and static and dynamic stabilizers) on the bearing surface throughout the range of motion; however, the benefits of robotic-assisted total knee arthroplasty (TKA) are debated.

**Objective:**

This quasi-randomized controlled trial (RCT) compares robotic-assisted and conventional TKA, focusing on surgical duration, hospital stay and serum markers. It aims to address current gaps in the literature and clarify potential advantages.

**Material and methods:**

All patients who received a TKA at the Department of Orthopedic Surgery of the Eifelklinik St. Brigida in Simmerath, Germany, between 2021 and 2025 were prospectively invited to participate in the present clinical trial. All patients followed the same clinical, imaging, and anesthesiological presurgical and postsurgical pathways irrespective of their allocation. All surgeries were performed using a standard medial parapatellar approach and a functional alignment philosophy. Both groups received the same implants, and patients followed the same postoperative physiotherapy program. Deviation from the planned surgical procedure and rehabilitation protocol warranted exclusion from the study. For patients allocated to robotic-assisted TKA, the CORI system (Smith & Nephew plc, Watford, United Kingdom) was used.

**Results:**

A total of 1099 patients completed the study, 59% (649 of 1099) of the patients were women and 50% (547 of 1099) of TKAs were performed on the left side. The mean body mass index (BMI) was 30.2 ± 4.9 kg/m^2,^ and the mean age was 66.9 ± 8.2 years. Comparability was found between the two cohorts regarding the number of women, side of surgery, mean BMI, age, hemoglobin, hematocrit and leucocyte count at admission. Robotic-assisted TKA was associated with a longer surgical time of 1.6 min (*p* = 0.04) and a lower C‑reactive protein level at both the first (*p* = 0.0003) and fifth (*p* = 0.003) postoperative days. No other difference between groups was found.

**Conclusion:**

Robotic-assisted TKA was associated with lower serum C‑reactive protein levels. No difference was found in the length of hospitalization and erythropoietic function in serum. Although the surgical execution of conventional TKA was statistically significantly faster, the clinical relevance of the endpoint surgical duration is negligible.

## Abstract (de)

**Hintergrund:**

Die Einführung der Navigationstechnik in der Knieendoprothetik, gefolgt von der Robotik, zielt darauf ab, die Ausrichtung der Komponenten zu verbessern und die Kontrolle der Belastungskräfte (d. h. Gewicht, Schwerkraft sowie statische und dynamische Stabilisatoren) auf die Auflagefläche über den gesamten Bewegungsbereich zu verbessern. Die Vorteile der roboterassistierten Knietotalendoprothese (TEP) sind jedoch umstritten.

**Ziel:**

Diese quasi-randomisierte kontrollierte Studie (RCT) vergleicht die roboterassistierte mit der konventionellen TEP und konzentriert sich dabei auf die Operationsdauer, den Krankenhausaufenthalt und die Serummarker. Ziel ist es, aktuelle Lücken in der Literatur zu schließen und mögliche Vorteile zu klären.

**Material und Methoden:**

Alle Patienten, die zwischen 2021 und 2025 in der Abteilung für Orthopädische Chirurgie der Eifelklinik St. Brigida in Simmerath, Deutschland, eine TEP erhielten, wurden prospektiv zur Teilnahme an der vorliegenden klinischen Studie eingeladen. Alle Patienten wurden unabhängig von ihrer Randomisierung den gleichen klinischen, bildgebenden und anästhesiologischen Vor- und Nachbehandlungen unterzogen. Alle Operationen wurden mit einem standardmäßigen medialen parapatellaren Zugang und einer funktionellen Ausrichtungsphilosophie durchgeführt. Beide Gruppen erhielten die gleichen Implantate, und die Patienten nahmen am gleichen postoperativen Physiotherapieprogramm teil. Eine Abweichung vom geplanten chirurgischen Verfahren und Rehabilitationsprotokoll führte zum Ausschluss aus der Studie. Bei den Patienten, die der roboterassistierten Chirurgie zugeteilt waren, wurde das CORI-System (Smith & Nephew plc, Watford, Vereinigtes Königreich) verwendet

**Ergebnisse:**

Insgesamt 1099 Patienten schlossen die Studie ab, 59 % (649 von 1099) waren Frauen und 50 % (547 von 1099) der Knie-TEP wurden auf der linken Seite durchgeführt. Der mittlere Body-Mass-Index (BMI) lag bei 30,2 ± 4,9 kg/m^2^, und das Durchschnittsalter betrug 66,9 ± 8,2 Jahre. Die beiden Kohorten waren in Bezug auf die Anzahl weiblicher Patienten, die Operationsseite, den mittleren BMI, Alter, Hämoglobin, Hämatokrit und Leukozytenzahl bei der Aufnahme vergleichbar. Die roboterassistierte TEP war mit einer längeren Operationszeit von 1,6 min (*p* = 0,04) und einem niedrigeren Werten des C‑reaktiven Proteins (CRP) sowohl am ersten (*p* = 0,0003) als auch am fünften (*p* = 0,003) postoperativen Tag verbunden. Es wurde kein weiterer Unterschied zwischen den Gruppen festgestellt.

**Schlussfolgerung:**

Die roboterassistierte TEP war mit niedrigeren CRP-Werten im Serum verbunden. Hinsichtlich der Dauer des Krankenhausaufenthalts und der erythropoetischen Funktion im Serum wurde kein Unterschied festgestellt. Obwohl die chirurgische Durchführung der konventionellen TEP statistisch signifikant schneller war, ist die klinische Relevanz des Endpunkts Operationsdauer vernachlässigbar.

## Introduction

Knee osteoarthritis is common [1, 2]. In selected patients with symptomatic and severe osteoarthritis, total knee arthroplasty (TKA) may be necessary [3–6], aiming to restore the native knee function and improve the quality of life and daily activities [7]. The TKA is associated with good functional outcomes and a low rate of complications [3, 8, 9]. It has undergone significant evolution over the past few decades, with improvements in biomaterials and implants [10, 11]. Furthermore, various surgical approaches have been introduced, differing in invasiveness (e.g., quad-sparing, mid-vastus and sub-vastus), concepts of alignment (mechanical, kinematic, restricted kinematic, and functional), gap balancing and postoperative rehabilitation protocols [4, 6, 8, 12]. Despite continuous progress, persistent pain after TKA remains a concern [13, 14], with the prevalence of chronic pain at 3–24 months postoperatively being at least 20% [15–18]. In most patients no cause for such persistent pain is identified, although malpositioning of components is a common underlying cause [19, 20]. The advent of navigation, followed by robotics in knee prosthetic surgery aims, among other things, to enhance the alignment of components and to improve the control of stress forces (i.e., weight, gravity, and static and dynamic stabilizers) on the bearing surface throughout the range of motion [21, 22]; however, the clinical implications of functional alignment in robotic-assisted TKA are still debated [23]. Despite numerous studies and consensus meetings, the advantages of robotic-assisted TKA have not been fully clarified [23] and gaps in the current literature persist. Whether differences in serum inflammatory and erythropoietic function markers exist is still unclear [24, 25]. In addition, whether robotic-assisted TKA is associated with a shorter hospitalization than conventional TKA remains unclear [8, 23]. Therefore, a quasi-randomized controlled trial (RCT) was conducted to clarify these gaps, comparing robotic-assisted TKA versus conventional freehand TKA. The outcomes of interest were the duration of surgery, length of hospitalization, inflammation and erythropoietic function markers.

## Methods

### Study design

All patients who underwent a TKA at the Department of Orthopaedic Surgery of the Eifelklinik St. Brigida in Simmerath (Germany) from 2021 to 2025 were prospectively invited to participate in this clinical trial. The institution where the surgeries are performed is accredited by “EndoCert” (EndoCert certificate, Centers of German Endoprosthetics, German Society for Orthopaedics and Traumatology) [26], which supervises and certifies the quality of the surgical procedures. This study was conducted in accordance with the principles of the Declaration of Helsinki and its subsequent amendments. The study protocol was prospectively registered and approved by the German Registry of Clinical Trials (ID DRKS00030614). Ethics approval was granted by the North Rhine Medical Council, Düsseldorf, Germany (ID 2022374). The protocol of the present quasi-RCT was previously published [22]. For patients allocated to robotic-assisted TKA, the CORI system (Smith & Nephew plc, Watford, United Kingdom) was used.

### Randomization and blinding

Patients who consented to participate in the current study were preoperatively informed of its purposes and signed a written informed consent form to confirm their willingness to take part in the trial. Enrolment in the study did not influence or alter the standards used to manage patients at our institution. This study is a single-blind parallel-group quasi-RCT. Patients were randomly assigned to either robotic-assisted TKA or conventional freehand TKA. All patients for whom TKA was indicated were sequentially allocated in a 1:1 ratio to surgeons who performed robotic-assisted TKA or those who performed conventional freehand TKA during their outpatient appointment. Patients remained blinded to the allocation until the first postoperative day (POD). Surgeons and personnel involved in the clinical management of the patients were not blinded to the allocation.

### Eligibility criteria

The inclusion criteria were: (1) age over 18 years, (2) ability to consent, (3) symptomatic knee osteoarthritis stages II–IV according to the Kellgren-Lawrence classification. The exclusion criteria were: (1) acute or chronic inflammatory diseases, (2) neoplastic diseases, (3) pregnancy and lactation, (4) uncontrolled coagulopathy, (5) abnormal cell count, (6) severe peripheral neuropathy, (7) vascular diseases, (8) peripheral ulcers, (9) missing data on the endpoint of interest, (10) blood tests taken on POD other than 1 and 5, (11) patients unable to adhere to the postoperative management protocol, (12) other conditions that might have influenced the results of the present study.

### Surgical technique

All patients followed the same clinical, imaging and anesthesiological preoperative and postoperative pathways, irrespective of their allocation. Each patient received a 1.5 g single administration of intravenous cefuroxime at the induction of general anesthesia. A continuous femoral nerve block was used for pain control and maintained for 48 h. All surgeries were conducted using a standard medial parapatellar approach and a restricted kinematic alignment following the principles of the coronal plane alignment of the knee (CPAK) classification [27]. All components were implanted in accordance with the manufacturer’s instructions using the Smith & Nephew Legion Genesis II (Smith & Nephew plc, Watford, United Kingdom), with a posterior stabilized polyethylene liner insert. Both femoral and tibial implants were cemented using Palacos cement (Heraeus Medical GmbH, Wehrheim, Germany). At the conclusion of the procedure, 1 g of tranexamic acid was injected intra-articularly, one closed suction deep drain and one open suction subcutaneous drain were employed for the first 48 h. Antithrombotic prophylaxis with enoxaparin sodium (40 mg/0.4 ml daily, subcutaneously) for 6 weeks, was initiated 12 h after the index procedure. Physiotherapy adhered to standard protocols [28]. A team of physiotherapists attended to patients during hospitalization from the first POD. In the absence of complications or other medical reasons that prevent discharge, the minimum length of hospitalization at our institution is 5 days. In cases of postoperative complications or delayed functional recovery, such as limited knee flexion or extension, impaired ambulation, difficulty performing physiotherapy exercises or inability to perform a straight leg raise, hospitalization is extended beyond the minimum 5‑day stay. Furthermore, from the second POD, each patient underwent two daily physiotherapy sessions utilizing continuous passive motion for 60 min to flex and extend the knee joint. The physiotherapist progressively increased the range of motion at each session. Patients were discharged when they achieved at least 80° of flexion. Starting from POD 2, patients began walking under physiotherapist supervision, and on POD 4, they started ascending and descending stairs. A personalized outpatient or inpatient rehabilitation program was established for each patient, lasting a minimum of 3 weeks. Deviation from the planned surgical procedure and rehabilitation protocol warranted exclusion from the study.

### Outcomes of interest

Surgeons who used robotic-assisted TKA had performed at least 50 procedures before beginning this study. Data on surgical duration and length of hospitalization were collected. Upon admission and at POD 5, the following data were collected: inflammation markers, e.g., C‑reactive protein (CRP) and leucocyte counts and erythropoietic function markers (hemoglobin and hematocrit). The duration of surgery was measured from the beginning of the skin incision to the completion of the wound suture.

### Statistical analysis

All statistical analyses were performed using the software IBM SPSS version 25 (IBM, Armonk, NY, USA). Continuous data were analyzed using the mean difference (MD), while the odds ratio (OR) effect measures were calculated for dichotomic data. The confidence interval (CI) was set at 95% for all comparisons. The T‑test and *χ*^2^-tests were performed with values of *P* < 0.05 considered statistically significant. A post hoc power analysis was conducted to assess the statistical power for detecting differences in CRP levels between groups. Based on the observed means and standard deviations (SD), Cohen’s d was calculated for CRP at POD 1 and POD 5. Using a significance level of α = 0.05 and a sample size of 500 per group, the power was estimated for each comparison to evaluate the adequacy of the study design for these endpoints.

## Results

### Recruitment process

**Fig. 1:**
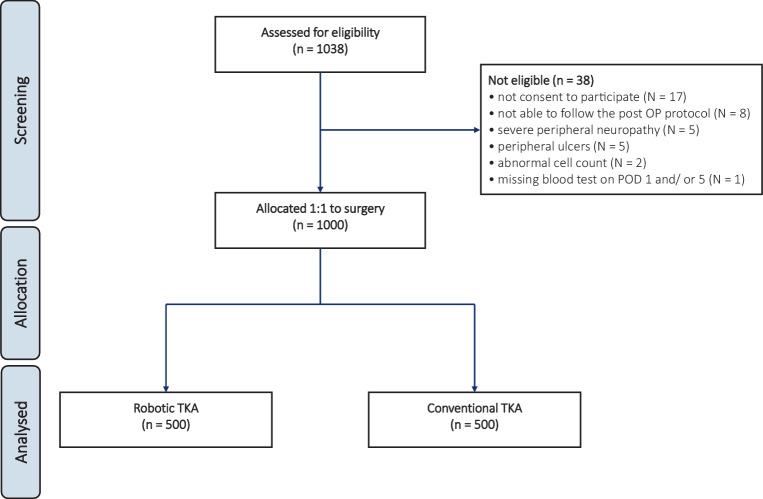
CONSORT diagram of the recruitment process

A total of 1038 patients were initially recruited. Of these, 38 were deemed ineligible due to lack of consent to participate (*N* = 17), inability to follow the postoperative protocol (*N* = 8), severe peripheral neuropathy (*N* = 5), peripheral ulcers (*N* = 5), abnormal cell count (*N* = 2) and blood tests not taken on POD 1 or 5 (*N* = 1). Ultimately, 1000 patients underwent surgery: 500 were allocated to robotic-assisted TKA and 500 to conventional freehand TKA (Fig. 1).

### Patient demographics

Data from 1000 patients (500 patients in each group) are reported of which 65% (649 of 1000 patients) were women and 55% (547 of 1000) of TKAs were performed on the left side. The mean body mass index (BMI) was 30.2 ± 4.9 kg/m^2,^ and the mean age was 66.9 ± 8.2 years. Comparability was found between the two cohorts in the number of women, side of surgery, mean BMI, age, hemoglobin, hematocrit and leucocyte counts (Table 1).

**Table 1:** Baseline comparability

Endpoint	Robotic (*N* = 500)	Conventional (*N* = 500)	*P*
Women (*n*)	54% (252 of 500)	80% (400 of 500)	0.09
Side (*left*)	44% (222 of 500)	65% (327 of 500)	0.1
Body mass index (k*g/m*^*2*^)	30.0 ± 4.6	30.2 ± 5.1	0.6
Age (*years*)	66.7 ± 8.4	67.0 ± 8.1	0.6
Hemoglobin (*g/dl*)	14.3 ± 1.3	14.3 ± 1.5	0.7
Hematocrit (*%*)	42.7 ± 4.1	43.0 ± 4.6	0.3
Leucocytes (*x10*^*3*^*/µl*)	7.0 ± 1.6	7.0 ± 1.8	0.9

### Outcomes of interest

Robotic-assisted TKA was associated with longer surgical time (MD 1.6 min; *p* = 0.04) and a lower CRP at POD 1 (MD −0.7; *p* = 0.0003) and POD 5 (MD −0.7; *p* = 0.003). No other differences between groups was found (Table 2).

**Table 2:** Outcomes of interest

Endpoint		Robotic (*N* = 500)	Conventional (*N* = 500)	MD	*P*
Surgical duration (min)		82.9 ± 16.6	81.3 ± 20.1	1.6	0.04
Hospitalization (*days*)		6.2 ± 1.9	6.1 ± 1.8	0.1	0.3
POD 1	CRP (*mg/dl*)	3.5 ± 3.2	4.2 ± 2.6	−0.7	0.0003
	Hemoglobin (*g/dl*)	11.8 ± 2.2	11.8 ± 1.4	0.0	1.0
	Hematocrit (*%*)	35.5 ± 5.3	35.3 ± 4.1	0.2	0.5
	Leucocytes (*x10*^*3*^*/µl*)	10.7 ± 5.6	10.4 ± 2.5	0.3	0.2
POD 5	CRP (*mg/dl*)	7.8 ± 4.6	8.5 ± 4.6	−0.7	0.003
	Hemoglobin (*g/dl*)	10.8 ± 1.5	10.9 ± 1.8	−0.1	0.4
	Hematocrit (*%*)	32.6 ± 5.6	32.4 ± 4.6	0.1	0.7
	Leucocytes (*x10*^*3*^*/µl*)	7.3 ± 1.9	7.3 ± 1.8	0.0	0.9

*POD* postoperative day, *CRP* C-reactive protein

### Post hoc power analysis

A post hoc power analysis was performed for CRP levels at POD 1 and POD 5, yielding Cohen’s effect sizes of 0.24 and 0.15, respectively, with a significance level set at α = 0.05. These values indicate adequate statistical power to detect the observed differences, particularly at POD 1, supporting the reliability of the inflammatory outcome measures.

## Discussion

According to the main findings of the present investigation, robotic-assisted TKA was associated with longer surgical times and lower serum CRP at POD 1 and POD 5. No difference was found in the length of hospitalization and erythropoietic function in serum. Although conventional TKA was statistically significantly faster, the clinical relevance of the endpoint surgical duration is uncertain. Indeed, a mean difference of 1.6 min over an approximately 82-min surgical intervention is negligible and of no clinical relevance.

The relevance of lower CRP observed in the robotic-assisted TKA is not fully clear: CRP is a widely recognized acute-phase reactant synthesized by the liver in response to proinflammatory cytokines, particularly interleukin 6 (IL-6) [29, 30]. Its serum concentration increases rapidly following surgical trauma, infection or tissue injury and it is routinely used as a biomarker to monitor postoperative inflammatory responses, including those following TKA [31, 32]. Typically, CRP levels rise sharply after TKA, peaking within 48–72 h postoperatively and declining over the following 7–14 days in uncomplicated cases [33, 34]. This difference in CRP might follow the different modality of anatomical axis alignment of the lower limb [35]. In conventional TKA, we use intramedullary alignment for the femoral and tibial anatomic axes at our institution. The use of intramedullary instrumentation during TKA introduces an additional component of bone and marrow trauma [36, 37]. This intramedullary violation may contribute to a more pronounced systemic inflammatory response from the mechanical disruption of the medullary canal, marrow contents and associated vasculature [38]. As a result CRP levels may be marginally elevated or prolonged in patients undergoing TKA with intramedullary guides compared to those in whom extramedullary (EM) alignment systems are employed [39, 40]. The physiological rationale behind this observation lies in the direct mechanical insult to the endosteal surfaces and bone marrow, a potent stimulus for releasing proinflammatory mediators [41, 42]. Furthermore, reaming or inserting alignment rods or nails into the intramedullary canal can lead to embolization of marrow contents into the systemic circulation, potentially amplifying the inflammatory cascade [43, 44]. Although these processes are typically well-tolerated in healthy individuals, they may contribute to variations in the magnitude and duration of CRP elevation in the early postoperative period [45, 46]; however, it is essential to contextualize the clinical relevance of these CRP changes. In most patients, CRP levels remain within the expected physiological range for TKA, even with intramedullary instrumentation and follow a predictable temporal decline [47, 48]. Thus, while intramedullary techniques may modestly influence the CRP trajectory, this should not be misconstrued as a pathological finding without other clinical or laboratory indicators of complications [49, 50]. Persistent CRP elevation beyond the second postoperative week, secondary spikes or failure to decline appropriately should prompt further investigation [51, 52]. The lower postoperative CRP levels observed in the robotic-assisted TKA group may be explained by the reduced surgical trauma associated with robotic assistance [53, 54]. Robotic systems enable more precise bone resections and soft tissue handling, minimizing unnecessary disruption of surrounding structures [55]. This targeted approach likely reduces local tissue injury and the subsequent systemic inflammatory response, reflected in lower CRP levels [56]. Additionally, robotic techniques often involve optimized surgical planning and may limit intraoperative microtrauma, further attenuating the acute phase response. The selective reduction in CRP, without differences in other blood variables or hospital stay, suggests that robotic-assisted TKA may offer localized biological advantages without affecting broader clinical outcomes.

The present study is a quasi-RCT in which patients were allocated 1:1 to a group based on the chronological order of their reservation. This modality might introduce a risk of selection bias and should be considered when interpreting the results. No difference was found in the length of the hospitalization. At our department, patients are admitted for a minimum of 5 days. At POD 5, patients underwent radiographs of the lower limb and knee in a lateral projection as well as blood tests. If patients have reached at least 80 ° of flexion and have no wound healing problems or other complications, they are discharged to an inpatient physiotherapist. This minimum hospitalization of 5 days might underpower differences in the length, representing a possible limitation of the present clinical trial.

## Conclusion

Robotic-assisted TKA was associated with lower serum CRP at POD 1 and POD 5. No difference was found in the length of hospitalization and serum erythropoietic function. Although conventional TKA was statistically significantly faster, the clinical relevance of the endpoint surgical duration is probably negligible.

## Data Availability

The datasets generated during and/or analyzed during the current study are available throughout the manuscript.

## Abbreviations

BMI: Body mass index
CI: Confidence interval
CPAK: Coronal plane alignment of the knee
CRP: C‑reactive protein
MD: Mean difference
OR: Odds ratio
POD: Postoperative day
RCT: Randomised controlled trial
SD: Standard deviations
TKA: Total knee arthroplasty

## Declarations

Ethics approval was granted by the North Rhine Medical Council, Düsseldorf, Germany (ID 2022374). Registration: German Registry of Clinical Trials (ID DRKS00030614). All patients provided written consent to use their clinical and imaging data for research purposes.
